# Nobiletin restores HFD-induced enteric nerve injury by regulating enteric glial activation and the GDNF/AKT/FOXO3a/P21 pathway

**DOI:** 10.1186/s10020-024-00841-8

**Published:** 2024-08-02

**Authors:** Yueshan Pang, Li Zhang, Zhuoting Zhong, Ni Yang, Yali Zheng, Weijun Ding

**Affiliations:** 1 Department of Fundamental Medicine, Chengdu University of Traditional Chinese Medicine, Chengdu, 611130 China; 2 The Second Clinical Medical College, North Sichuan Medical College, Nanchong Central Hospital, Nanchong, 637000 China

**Keywords:** Nobiletin, Enteric nerve, High-fat diet (HFD), GDNF/AKT/FOXO3a pathway, Trem2

## Abstract

**Background:**

To explore whether nobiletin has a protective effect on high-fat diet (HFD)-induced enteric nerve injury and its underlying mechanism.

**Methods:**

An obesity model was induced by a HFD. Nobiletin (100 mg/kg and 200 mg/kg) and vehicle were administered by gastric gavage for 4 weeks. Lee’s index, body weight, OGTT and intestinal propulsion assays were performed before sacrifice. After sampling, lipids were detected using Bodipy 493/503; lipid peroxidation was detected using MDA and SOD kits and the expression of PGP 9.5, Trem2, GFAP, β-tubulin 3, Bax, Bcl2, Nestin, P75 NTR, SOX10 and EDU was detected using immunofluorescence. The GDNF, p-AKT, AKT, p-FOXO3a, FOXO3a and P21 proteins were detected using western blotting. The relative mRNA expression levels of NOS2 were detected via qPCR. Primary enteric neural stem cells (ENSCs) were cultured. After ENSCs were treated with palmitic acid (PA) and nobiletin, CCK-8 and caspase-3/7 activity assays were performed to evaluate proliferation and apoptosis.

**Results:**

HFD consumption caused colon lipid accumulation and peroxidation, induced enteric nerve damage and caused intestinal motor dysfunction. However, nobiletin reduced lipid accumulation and peroxidation in the colon; promoted Trem2, β-tubulin 3, Nestin, P75NTR, SOX10 and Bcl2 expression; inhibited Bax and GFAP expression; reduced NOS2 mRNA transcription; and regulated the GDNF/AKT/FOXO3a/P21 pathway. Nobiletin also promoted PA-induced impairment of ENSCs.

**Conclusions:**

Nobiletin restored HFD-induced enteric nerve injury, which may be associated with inhibiting enteric nerve apoptosis, promoting enteric nerve survival and regulating the GDNF/AKT/FOXO3a/P21 pathway.

**Supplementary Information:**

The online version contains supplementary material available at 10.1186/s10020-024-00841-8.

## Background

Obesity has become a prominent public health problem worldwide. A high-fat diet (HFD) is one of the main causes of obesity. A HFD can cause damage to the nervous system (Muscat et al. 2023). The induction of enteric nerve damage by a HFD has attracted increasing attention in recent years (Almeida et al. 2022). Dou et al. (2020) suggested that a HFD induces a decrease in the number of enteric nerves, especially cholinergic neurons, in the enteric nervous system. A HFD also induces the activation of glial cells and thus increases colonic tachykinin-induced contraction (D'Antongiovanni et al. 2020; Antonioli et al. 2020), decreases PGP9.5 and nNOS expression, and disrupts intestinal motor function in HFD-induced obese mice (Bhattarai et al. 2016; Pang et al. 2022). In conclusion, HFD induce enteric nerve damage, but there are still relatively few therapeutic drugs for HFD-induced enteric nerve damage. Therefore, finding an effective therapeutic drug for HFD-induced enteric nerve damage is very important.

Nobiletin is a polymethoxyflavone extracted from orange peel that has various biological functions, such as anti-inflammatory (Adhikari-Devkota et al. 2019) and antioxidant effects (Lee and Kim 2022), promotion of lipid metabolism (Li et al. 2022), regulation of blood glucose (Lai et al. 2022) and neuroprotection (Furukawa et al. 2021). Studies have suggested that nobiletin can protect hippocampal neurons through the cAMP/PKA/ERK/CREB pathway (Takito et al. 2016; Matsuzaki et al. 2006). Nobiletin reduced the expression of IL-1β, IL-6, TNF-α, Bax and caspase3 and promoted the expression of IL-10 through the TLR4/NF-κB and mitogen-activated protein kinase (MAPK) signalling pathways (Pang et al. 2022; Wang et al. 2019; Zheng et al. 2017). Nobiletin can reduce depressive symptoms by regulating the expression of brain-derived neurotrophic factor (BDNF), synaptic protein I and TrkB (Li et al. 2013) in the hippocampus. In addition, nobiletin can inhibit the activation of microglia, promote the expression of glial cell line-derived neurotrophic factor (GDNF), and alleviate neurodegeneration in Parkinson’s disease (Yang et al. 2017). In conclusion, nobiletin can inhibit neuroinflammation and apoptosis and promote the expression of neurotrophic factors in the central nervous system. However, there are few reports on the effects of nobiletin on the enteric nerve.

We designed this study to explore whether nobiletin has a protective effect on HFD-induced enteric nerve damage and to determine its underlying mechanism.

## Methods

### Animal studies

This study was approved by the Animal Ethics Committee of the Chengdu University of Traditional Chinese Medicine (No. 2021-05), and all experiments were performed in accordance with relevant guidelines and regulations. One hundred and six 8-week (w)-old male C57BL/6J mice were purchased from Sibeifu (Beijing, China) (certificate no. SCXK (Jing) 2019-0008). The mice were raised in laboratory cages under standard conditions (22–24 °C, 12-h light and dark cycle), with no restrictions on eating or drinking. Twelve mice were selected randomly and fed a control diet (D12450J, Research Diets, USA) (CD group), and the remaining mice were fed a HFD (D12492, Research Diets, USA) for 8 w to establish a diet-induced obesity model. According to the criteria for identifying obese mice (Dou et al. 2020), fifty-six obese mice were obtained. Based on our previous experience (Pang et al. 2022), obese mice were randomized equally into the HFD group, middle-dose nobiletin treatment group (NOBM) (100 mg/kg/d) and high-dose nobiletin treatment group (NOBH) (200 mg/kg/d). Nobiletin (Ruifensi Biotech Co., Ltd., Chengdu, China) (purity > 98%) was suspended in 0.5% sodium carboxymethylcellulose and administered by gastric gavage for 4 w. A sterile saline vehicle was given to the CD and HFD groups. Mice were sacrificed at 17 w, 18 w and 20 w, and samples were taken.

### Whole gut transit detection

The mice were deprived of food and given water. Then, the mice were gavaged with 0.2 ml of semisolid paste (10 g of carboxymethylcellulose, 16 g of whole milk powder, 8 g of sugar and 2 g of active charcoal were dissolved in 300 ml of deionized water). Individual mice were placed in a cage and in a quiet environment. The frequency of defecation within 8 h (h) was recorded for statistical analysis.

### Fasting blood glucose and oral glucose tolerance test (OGTT)

After a 12 h overnight fast, the fasting blood glucose was tested at 16 w. Additional plasma glucose was checked at 0 min, 30 min, 60 min, 90 min and 120 min for the OGTT after administration of 2 g/kg glucose at the end of 20 w. The plasma glucose was calculated using the area under the curve (AUC) method.

### Hematoxylin and eosin (HE) staining and neuropathological scoring

Colon neuropathology was assessed by hematoxylin and eosin (HE) staining. An HE staining kit (C0105S) was purchased from Biyuntian Biotechnology (Shanghai, China). In accordance with the manufacturer's instructions, HE staining was performed, and light microscope images were taken of the tissue sections. According to our previous study (Dou et al. 2020; Pang et al. 2022). neuropathological scoring was performed. The score was divided into five grades. No damage = 0 points. At mild = 1 point, there was only a low degree of nerve cell edema and degeneration. At moderate = 2 points, the nerve fibres exhibited severe oedema, and the space was widened. At severe = 3 points, the nerve fibres exhibited severe oedema, and the cell nuclei exhibited deep staining, an irregular morphology and a disorganized arrangement. At extremely severe = 4 points, the number of neuronal cell nuclei varied in size, and the cell morphology was irregular. Four visual fields (upper, lower, left and right) were scored, and the average of the data per slide was used for statistical analyses.

### Determination of lipid peroxidation

A superoxide dismutase (SOD) assay kit (A001-3-2, Jiancheng, Nanjing, China) and malondialdehyde (MDA) assay kit (A003-1-2, Jiancheng, Nanjing, China) were used to estimate liver and colon peroxidation. According to the instructions, the liver and colon were prepared as 10% tissue homogenates. The 100 μL supernatant of the tissue homogenate was used for lipid peroxidation assays.

### Fluorescence quantitative polymerase chain reaction (qPCR)

TRIzol (Invitrogen, Carlsbad, CA, USA) was used to extract RNA from colon tissues. RNA was reverse-transcribed into cDNA using SuperMix (Vazyme Biotech Co., Ltd., Nanjing,China). Some target genes were amplified and quantitatively analysed with the SYBR Premix EX Taq™ kit (Vazyme Biotech Co., Ltd., Nanjing,China) using qTOWER3 G (Analytik Jena, Jena, Germany). Supplementary Table 1 contains gene-specific primers designed and synthesized by FOREGENE (Chengdu, China). The relative expression of NOS2 mRNA was calculated by normalization to that of β-actin. To calculate fold changes, we used the 2^–ΔΔCt^ method.

### Immunofluorescence

The immunofluorescence method was performed according to our previous study (Pang et al. 2022). Primary antibodies against Trem2 (ab245227, rabbit), GFAP (ab4674, chicken), Bax (ab53154, rabbit), Bcl-2 (ab182858, rabbit), β-Tubulin3 (ab52623, rabbit), Nestin (ab81462, rat), SOX10 (ab212843, mouse), P75NTR (ab81462, rat) and PGP9.5 (ab52623, rabbit) were purchased from Abcam (Cambridge, MA, USA) and incubated at a 1:200 dilution. SOX2 (AF5140, rabbit) was obtained from Affinity Biosciences (Jiangsu, China) and incubated at a 1:200 dilution. The secondary antibody FITC-conjugated goat anti-rat IgG (E031240-01) was obtained from EarthOx Life Sciences (Millbrae, CA, USA). Donkey anti-chicken IgY H&L (FITC) (ab63507), goat anti-mouse IgG H&L (Alexa Fluor® 555) (ab150118) and goat anti-rabbit IgG H&L (Alexa Fluor® 647) (ab150083) were purchased from Abcam (Cambridge, MA, USA) and were used and diluted 1:1000. The sections were observed and photographed using fluorescence confocal microscopy (Leica, Italy). Four images were taken from the upper, lower, left and right portions of the section. The integrated density was quantified using ImageJ version 1.52 (ImageJ Software Inc., USA) for statistical analyses.

### 5-Ethynyl-2′-deoxyuridine (EDU) assay

The EDU (ST067) and BeyoClick™ EDU-647 Imaging Kit (C0081S) were purchased from Beyotime (Hangzhou, China). The mice in the HFD, NOBM and NOBH groups were injected with EDU (50 mg/kg/d weight, i.p.) at the start of treatment gavage. The proximal colon was collected after EDU i.p. 1 w and 2 w. The proximal colon was fixed in 4% paraformaldehyde for 24 h and dehydrated in a 20% and 30% sucrose solution for 24 h each. Then, the proximal colon was embedded at the optimum cutting temperature (− 20 °C) and cut into 10 μm sections. Then, 100 μL of click reaction solution was added to each section, and the sections were incubated at 37 °C for 30 min without illumination. The sections were then incubated with DAPI (C1006, Beyotime, Hangzhou, China) for 10 min (at 37 °C, without illumination) for nuclear staining. After washing, the sections were observed and photographed using the same method as described above. The integrated density was quantified using ImageJ version 1.52 (ImageJ Software Inc., USA) for statistical analyses.

### Bodipy 493/503 staining

The 10 μm thick frozen sections were prepared for the BODIPY assay. Bodipy 493/503 lipid dye (GC42959) was purchased from GLPBIO (GC42959, California, US) and was prepared at a final concentration of 2 μM in PBS. The frozen sections were incubated with 100 µL of Bodipy 493/503 working solution (2 μM) for 15 min (at 37 °C without illumination). After washing, the sections were observed and photographed using the same method as described above.

### Western blot (WB)

Total proteins from the proximal colon tissues were extracted using RIPA lysis buffer (P0013B, Beyotime, Hangzhou, China) containing a mixture of protease and phosphatase inhibitors. The protein concentration was measured using a BCA protein assay kit (P0009, Beyotime, Hangzhou, China). Proteins (30 μg) were separated using 10% SDS‒PAGE and then electrically transferred to PVDF membranes. The membranes were incubated with primary antibodies overnight at 4 °C. Primary antibodies against Akt (A17909, rabbit), p-Akt (A18675, rabbit), FOXO3a (A0102, rabbit), p-FOXO3a (AP0684, rabbit), P21 (A19094, rabbit), and β-actin (AC026, rabbit) were purchased from ABclonal (Wuhan, China) and diluted 1:2000. GDNF (ab18956, rabbit) was purchased from Abcam (Cambridge, MA, USA) and diluted 1:2000. The secondary antibody, goat anti-rabbit IgG (H + L) HRP (S0001) purchased from Affinity Biosciences (Jiangsu, China), was diluted 1:5000 and incubated at 37 °C for 2 h. The bands were developed with enhanced chemiluminescence substrate and visualized using Tanon GIS chassis control software v2.0 (Shanghai, China). The relative protein expression was used for statistical calculations and analysis.

### Primary enteric neural stem cell (ENSC) culture and intervention

According to a previous study (Gao et al. 2016) and further improvements, primary ENSCs were prepared from 18.5-day-old foetal mice (C57BL/6J). A single-cell suspension was prepared, counted, and plated into an uncoated T25 tissue culture flask (1 × 10^6^ cells/cm^2^) with ENSC culture medium. The ENSC culture medium was composed of DMEM/F12 (10565018, Invitrogen, Shanghai, China) supplemented with 2% B27 (17504044, Invitrogen, Shanghai, China) supplement, 1% N2 (17502048, Invitrogen, Shanghai, China), 20 ng/ml epidermal growth factor (PHG0311, Invitrogen, Shanghai, China), 20 ng/ml basic fibroblast growth factor (P09038, R&D Systems, Minnesota, USA) and 1% penicillin‒streptomycin (S110JV, BasalMedia, Shanghai, China). The culture flask was incubated in a humidified environment with 5% CO_2_ (37 °C). Half of the culture medium was replaced every 2–3 days. The ENSCs were harvested after 6–9 days for further investigation. Nobiletin was dissolved in DMSO and then diluted in ENSC culture medium to 20, 40, 80 and 100 μM for cellular toxicity detection. Palmitic acid (PA) was prepared as a palmitate and then diluted to 50, 100 and 200 μM with ENSC culture medium for cellular toxicity detection. Then, nobiletin (20, 40 and 80 μM) was added to the ENSC culture medium containing PA (200 μM). The following groups were used: C group (without PA and nobiletin), N20 group (with nobiletin at 20 μM), N40 group (with nobiletin at 40 μM), N80 group (with nobiletin at 80 μM), N100 group (with nobiletin at 100 μM), P200 + N20 group (with PA at 200 μM + nobiletin at 20 μM), PA200 + N40 group (with PA at 200 μM + nobiletin at 40 μM), and PA200 + N80 group (with PA at 200 μM + nobiletin at 80 μM).

### CCK-8 assay

The proliferation of ENSCs was measured using a CCK8 kit (abs50003, Absin, Shanghai, China). Single-cell suspensions of ENSCs were cultured in the corresponding medium at a density of 4 × 10^4^/ml. After incubation for 24 h, 48 h and 72 h, the CCK-8 assay was performed. Then, 10 µL of CCK8 was added to the wells and incubated at 37 °C for 5 h. The absorbance at 450 nm was detected. In each group, 3 replicates were prepared, and three repeat experiments were performed.

### Caspase3/7 activity assay

The apoptosis of ENSCs was measured using a caspase3/7 activity assay kit (abs50025, Absin, Shanghai, China). Supernatants of lysed ENSCs from each group were collected after centrifugation. The caspase3/7 reaction solution (50 µL) was added to the supernatant (50 µL) and incubated at 37 °C for 120 min. At 405 nm, the absorbance was detected. The enzyme activity of caspase3/7 was calculated from a standard calibration curve for statistical analysis. In each group, 4 replicates were prepared, and three repeat experiments were performed.

### Statistics

The results are expressed as the mean ± standard deviation (SD). The data were analysed using GraphPad Prism (V9.1.0). Normality was tested using the Shapiro‒Wilk test. The normality of the data distribution was tested using Student’s t test for pairwise comparisons. One-way ANOVA and Tukey’s test were used for multiple comparisons. A p value ≤ 0.05 was considered to indicate statistical significance.

## Results

### Nobiletin improved obesity-related indices and abnormal glucose tolerance in HFD-fed mice

**Fig. 1:**
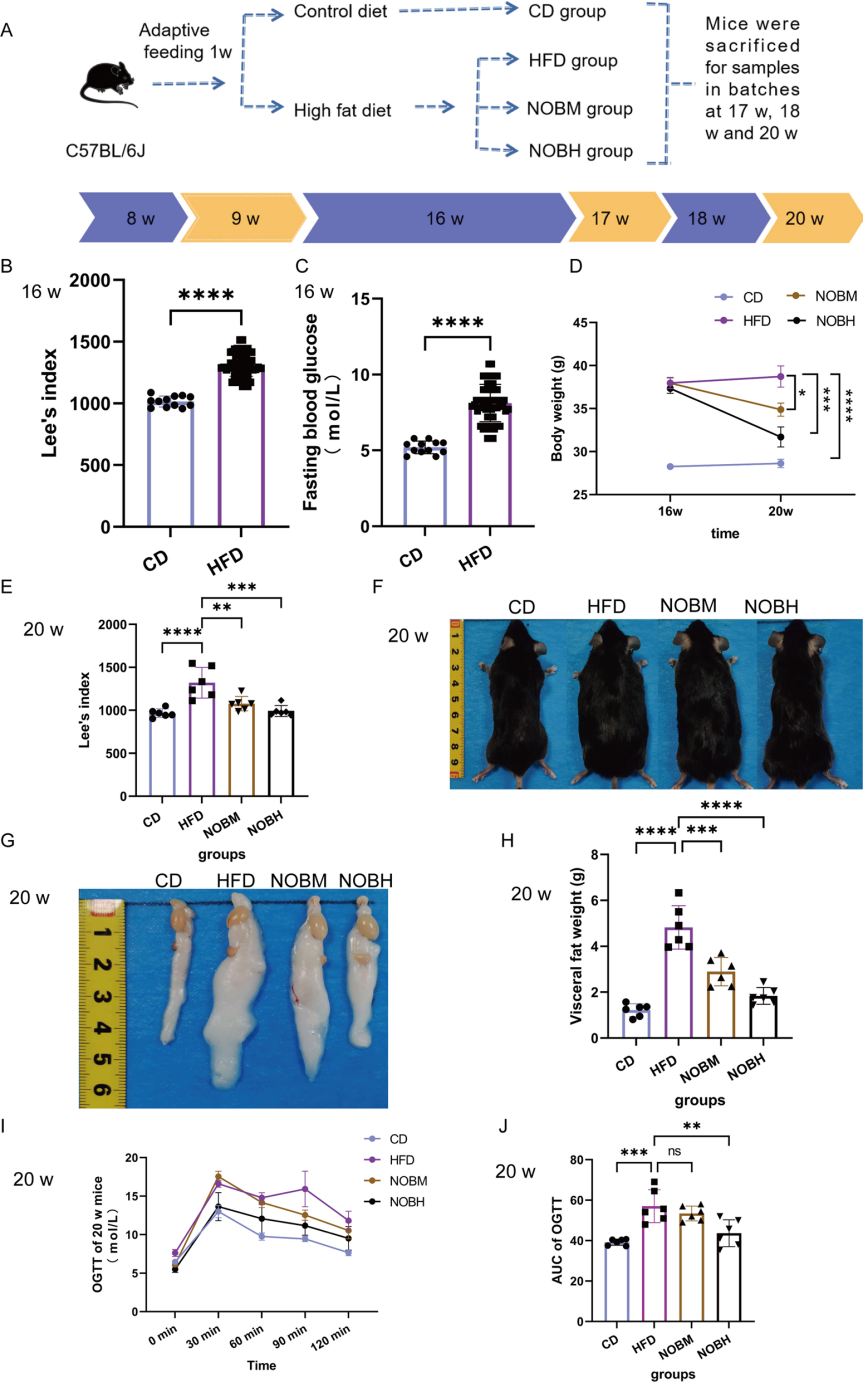
Nobiletin improved the obesity-related index and abnormal glucose tolerance in HFD-fed mice. **A** Timeline of the experimental protocol. **B** Lee’s index at 16 w. **C** Fasting glucose of 16 w. **D** Body weight. **E** Lee’s index at 20 w. **F** Body shape at 20 w. **G** Epididymal fat size at 20 w. **H** Visceral fat weight at 20 w (epididymal fat + mesenteric fat + perirenal fat). **I** OGTT at 20 w. **J** AUC of the OGTT. *P < 0.05, **P < 0.01, ***P < 0.001, ****P < 0.0001, ns > 0.05. **B**, **C** n = 12 (CD), n = 56 (HFD). **D**–**J** n = 6. CD: control group; HFD: high-fat diet group; NOBM, 100 mg/kg/d nobiletin; NOBH, 200 mg/kg/d nobiletin

Figure 1A illustrates the timeline of the experimental protocol. At 16 w, Lee's index (weight (g) ^ (1/3) × 10/body length (cm)) and the fasting blood glucose level were significantly greater in the HFD group than in the CD group (Fig. 1B and C) (P < 0.0001). These results suggested that obesity was successfully modelled. Combining fasting blood glucose data with our previous work on OGTT results at 16 w (Pang et al. 2022), HFD consumption induced glucose intolerance at 16 w. After treatment with nobiletin for 4 w, the body weight and Lee's index were significantly decreased compared with those of the HFD group (Fig. 1D and E) (P < 0.05 and 0.001). The mice in the NOBM and NOBH groups had a thinner body shape and smaller epididymal fat size than did those in the HFD group (Fig. 1F and G). The visceral fat content was significantly lower in the NOBM and NOBH groups than in the HFD group (Fig. 1H) (P < 0.001 and 0.0001). The AUC of the OGTT at 20 w was statistically analysed. Compared with that in the CD group, the extent of impaired glucose tolerance was greater in the HFD group (P < 0.001). However, the NOBH group showed a significant improvement in impaired glucose tolerance compared with the HFD group (Fig. 1I and J) (P < 0.01). These results indicated that nobiletin improved the obesity-related index and abnormal glucose tolerance in HFD-fed mice.

### Nobiletin decreased colonic lipid accumulation, peroxidation and neuropathological injury

**Fig. 2:**
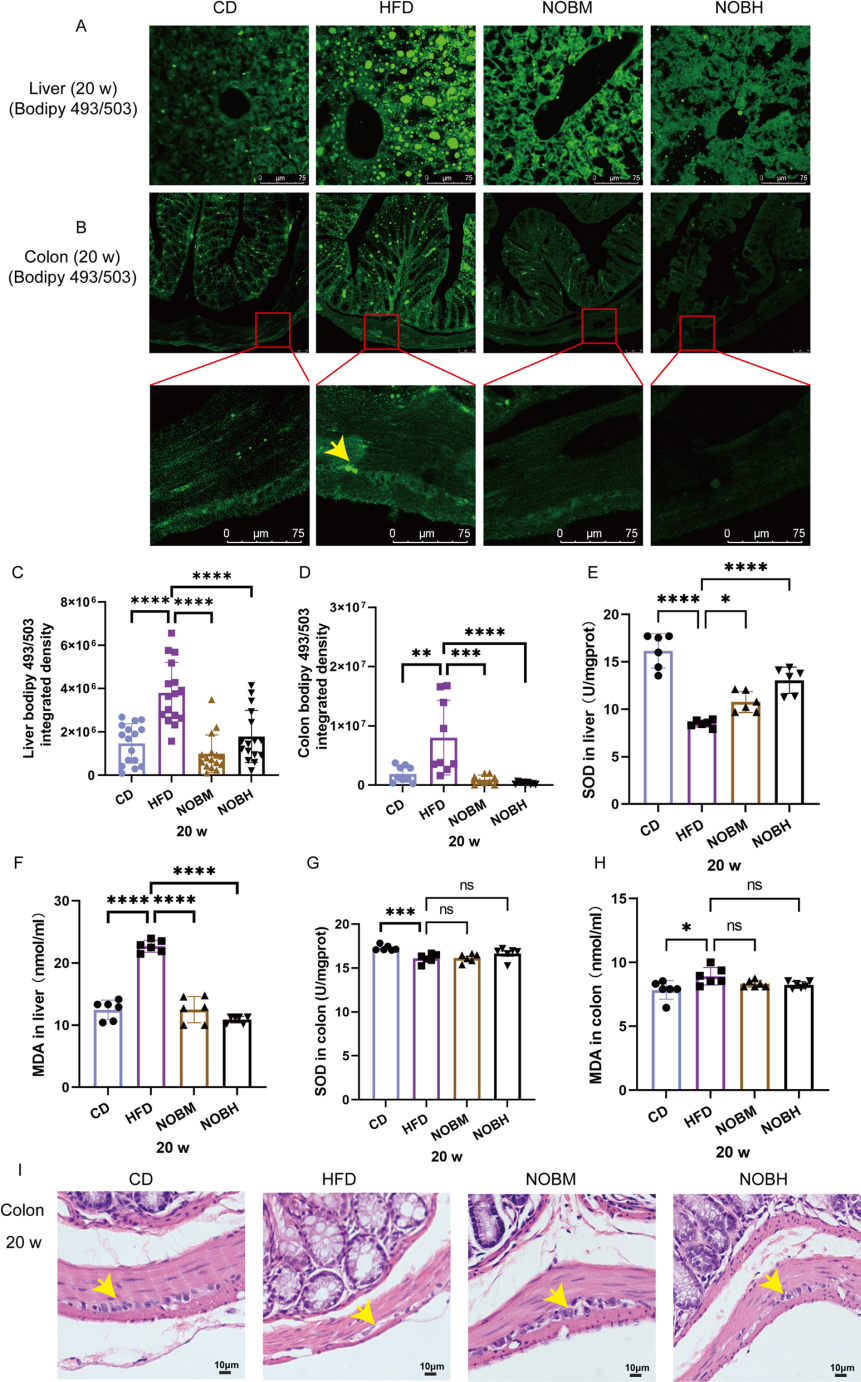
Nobiletin decreased lipid accumulation and peroxidation and alleviated colon neuropathologic damage in HFD mice. **A**, **B** Liver and colon lipid staining. **C**, **D** Statistical analyses of liver and colon lipids. **E**, **G** SOD activity in the liver and colon. **F**, **H** MDA content in the liver and colon. **I** HE staining. *P < 0.05, **P < 0.01, ***P < 0.001, ****P < 0.0001, ns > 0.05. n = 4, each dot in **C** and **D** represents the optical density of the images taken from the upper, lower, left and right portions of the section. CD: control group; HFD: high-fat diet group; NOBM, 100 mg/kg/d nobiletin; NOBH, 200 mg/kg/d nobiletin. Positive staining is indicated by a yellow arrow

A HFD induced a decrease in defecation frequency. However, nobiletin increased the defecation frequency in the NOBH group compared with that in the HFD group (Supplementary Fig. 1 A and B) (P < 0.01). Then, the accumulated lipids were detected in the colon and liver (Fig. 2A–D). The liver had more severe lipid deposits in the HFD group (Fig. 2A). Significant lipid deposits were also observed in the colonic mucosa, submucosa and ganglia in the HFD group (Fig. 2B). However, compared with those in the HFD group, the number of lipid deposits in the liver and colon was significantly reduced by nobiletin (Fig. 2A–D). A HFD induced lipid peroxidation (Liu et al. 2023). Then, MDA and SOD were detected in the liver and colon (Fig. 2E–H). Compared with the CD group, the SOD activity in the liver and colon was significantly decreased in the HFD group (Fig. 2E and G) (P < 0.0001 and 0.001). After treatment with nobiletin, SOD activity in the liver increased in the NOBM and NOBH groups (Fig. 2E and G) (P < 0.05 and 0.0001). MDA in the liver and colon was significantly greater in the HFD group than in the CD group (Fig. 2F, H) (P < 0.0001 and 0.05). However, nobiletin treatment decreased the MDA content in the liver (Fig. 2F and H) (all P < 0.0001) and colon, but the difference was not statistically significant in the colon (Fig. 2H). More severe pathological changes were observed in the HFD group (Fig. 2I, Table 1). However, nobiletin treatment significantly improved nerve injury caused by HFD (Fig. 2I, Table 1) (P < 0.0001 and 0.001). These results indicated that nobiletin reduced lipid deposits and peroxidation and alleviated colonic neuropathologic damage caused by a HFD.

**Table 1:** The neuropathologic scores of colonic tissues

Groups	Neuropathologic scores (mean ± SD)
CD	0.3584 ± 0.03627
HFD	2.176 ± 0.3045^a^
NOBM	1.695 ± 0.1442^b^
NOBH	1.825 ± 0.1429^c^

^a^Compared with the CD group, P < 0.0001; ^b^compared with the HFD group, P < 0.001; ^c^compared with the HFD group, P < 0.001

### Nobiletin restored HFD-induced enteric nerve damage by promoting Trem2 expression

**Fig. 3:**
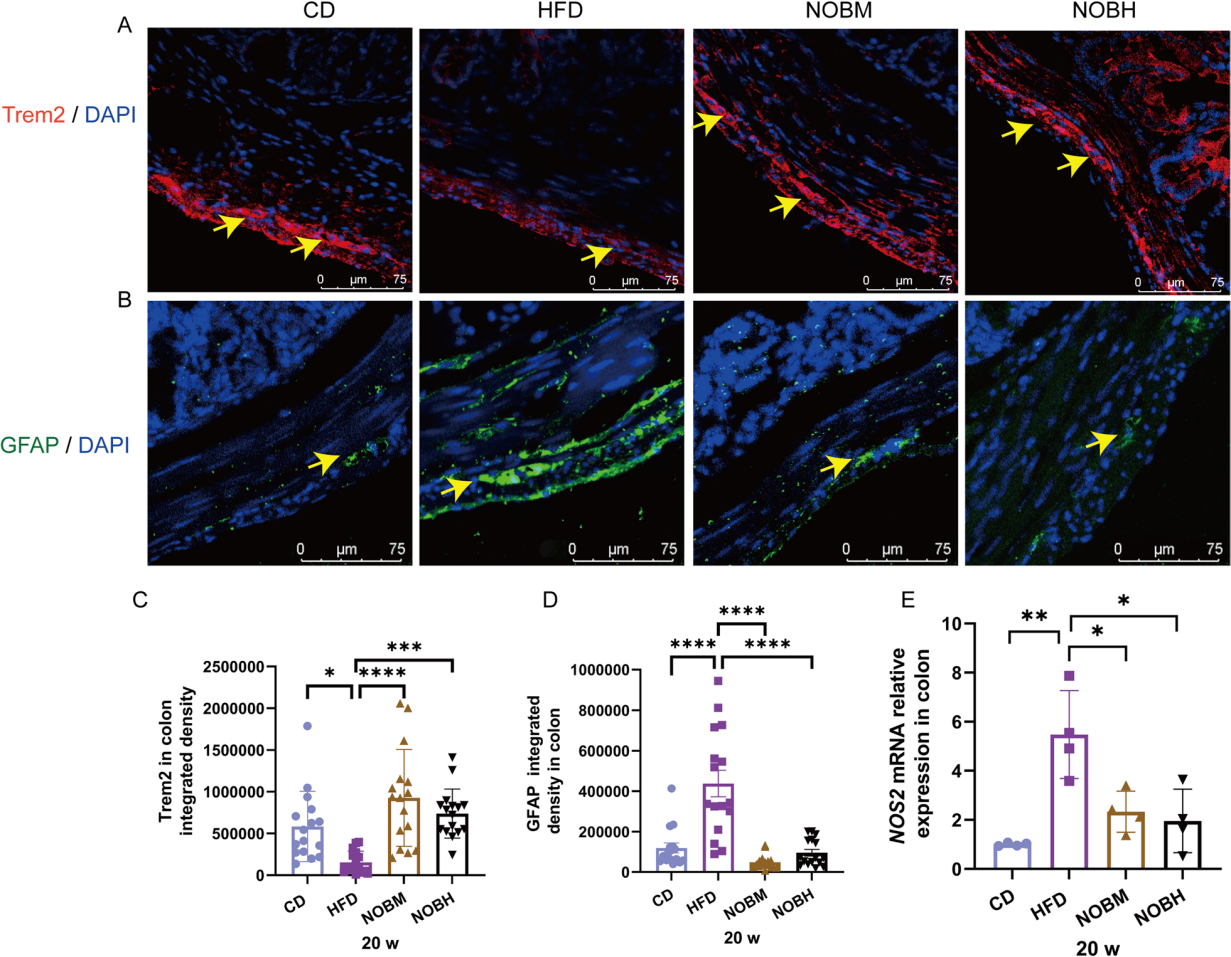
Nobiletin increased Trem2 expression and decreased enteric glial activation. **A**, **C** Trem2 expression and statistical analyses in the colon. **B**, **D** GFAP expression and statistical analyses in the colon. **E** Relative expression of NOS2 in the colon. *P < 0.05, **P < 0.01, ***P < 0.001, ****P < 0.0001. n = 4, each dot in **C** and **D** represents the optical density of the images taken from the upper, lower, left and right portions of the section. CD: control group; HFD: high-fat diet group; NOBM, 100 mg/kg/d nobiletin; NOBH, 200 mg/kg/d nobiletin. Positive staining is indicated by a yellow arrow

In our previous study (Pang et al. 2022), nobiletin was shown to regulate the expression of Trem2, which is associated with neuroinflammation (Pang et al. 2022). In this study, the Trem2 protein level was significantly lower in the HFD group than in the CD group (Fig. 3A and C) (P < 0.05). However, nobiletin treatment significantly promoted Trem2 protein expression (Fig. 3A and C) (P < 0.0001 and 0.001).The expression of GFAP, a marker of activated astrocytes, was significantly higher in the HFD group than in the CD group (Fig. 3B and D) (P < 0.0001). After treatment with nobiletin, GFAP was significantly decreased compared with that in the HFD group (Fig. 3B and D) (P < 0.0001). NOS2 is a marker of microglial proinflammatory activation, and relative mRNA expression was significantly increased in the HFD group (Fig. 3E) (P < 0.01). However, the relative NOS2 mRNA expression significantly decreased after treatment with nobiletin (Fig. 3E) (all P < 0.05). These results suggested that nobiletin reduced HFD-induced activation of enteric glia.

**Fig. 4:**
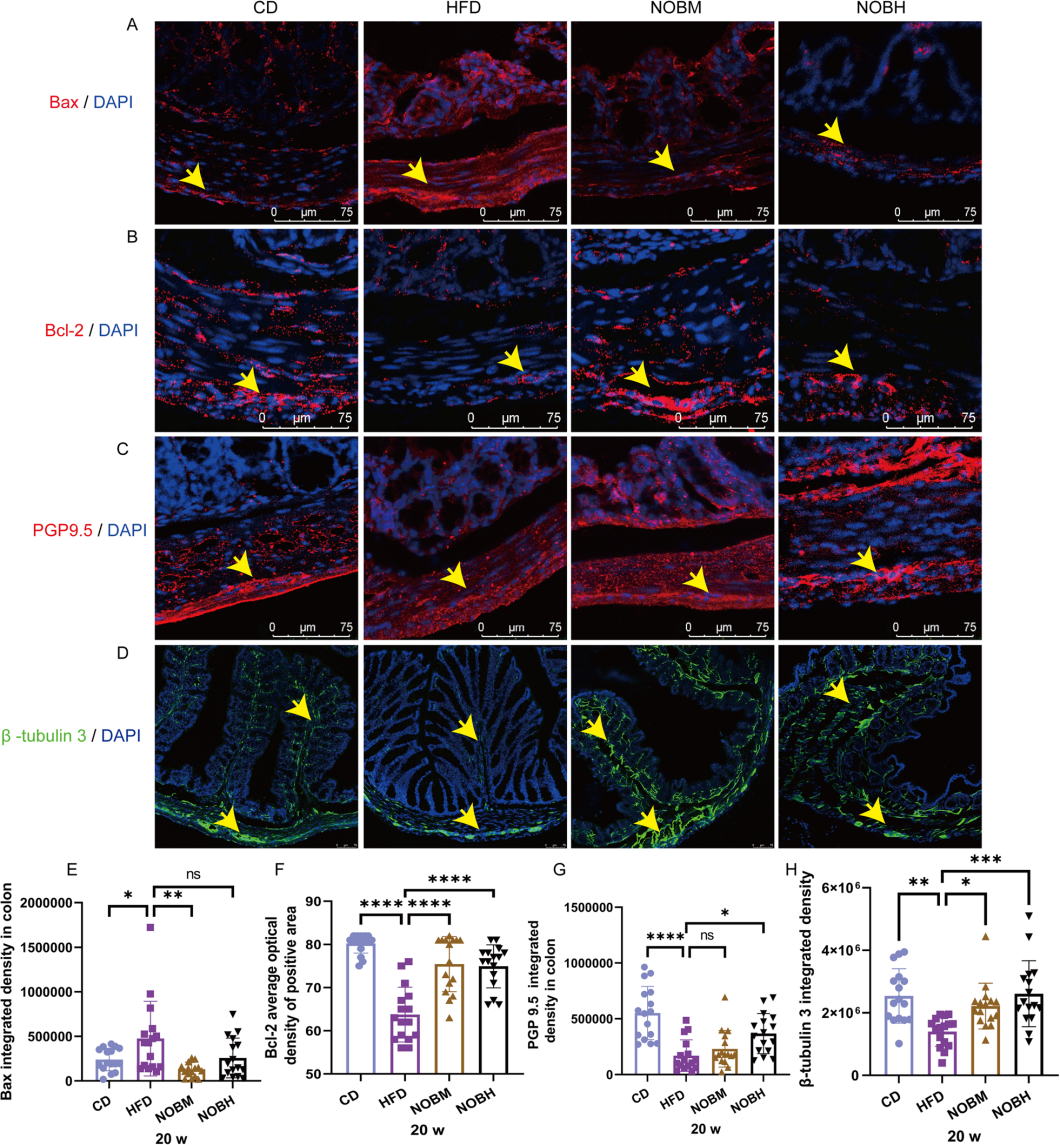
Nobiletin reversed HFD-induced enteric nerve damage. **A**, **E** Bax expression and statistical analyses. **B**, **F** Bcl-2 expression and statistical analyses. **C**, **G** PGP9.5 expression and statistical analyses. **D**, **H** β-Tubulin 3 expression and statistical analyses. *P < 0.05, **P < 0.01, ***P < 0.001, ****P < 0.0001, ns > 0.05. n = 4, each dot in **E**–**H** represents the optical density of the images taken from the upper, lower, left and right portions of the section. CD, control group; HFD, high-fat diet group; NOBM, 100 mg/kg/d nobiletin; NOBH, 200 mg/kg/d nobiletin. Positive staining is indicated by a yellow arrow

A HFD significantly increased Bax expression and decreased Bcl-2 expression in the colon (Fig. 4A, B, E, F) (P < 0.05 and 0.0001). However, nobiletin treatment significantly decreased Bax and increased Bcl-2 protein expression in the colon (Fig. 4A, B, E, F) (P < 0.01 and 0.0001). These results were consistent with the relative Bax and Bcl-2 mRNA expression observed in our previous studies (Pang et al. 2022). PGP9.5, a pan-neuronal marker, was significantly lower in the HFD group than in the CD group (Fig. 4C and G) (P < 0.0001). However, PGP9.5 in the NOBH group was significantly increased compared with that in the HFD group (Fig. 4C and G) (P < 0.05). β-Tubulin 3, a functional marker of neuritogenesis, was significantly decreased in the colonic mucosa and submucosa of the HFD group (Fig. 4D and H) (P < 0.01). However, nobiletin treatment significantly increased β-tubulin 3 expression (Fig. 4D and H) (P < 0.05 and 0.001). These results suggested that nobiletin reduced HFD-induced apoptosis and protected against HFD-induced neural injury.

### Nobiletin promoted enteric nerve regeneration and regulated the GDNF/AKT/FOXO3a/P21 pathway in HFD-fed mice

**Fig. 5:**
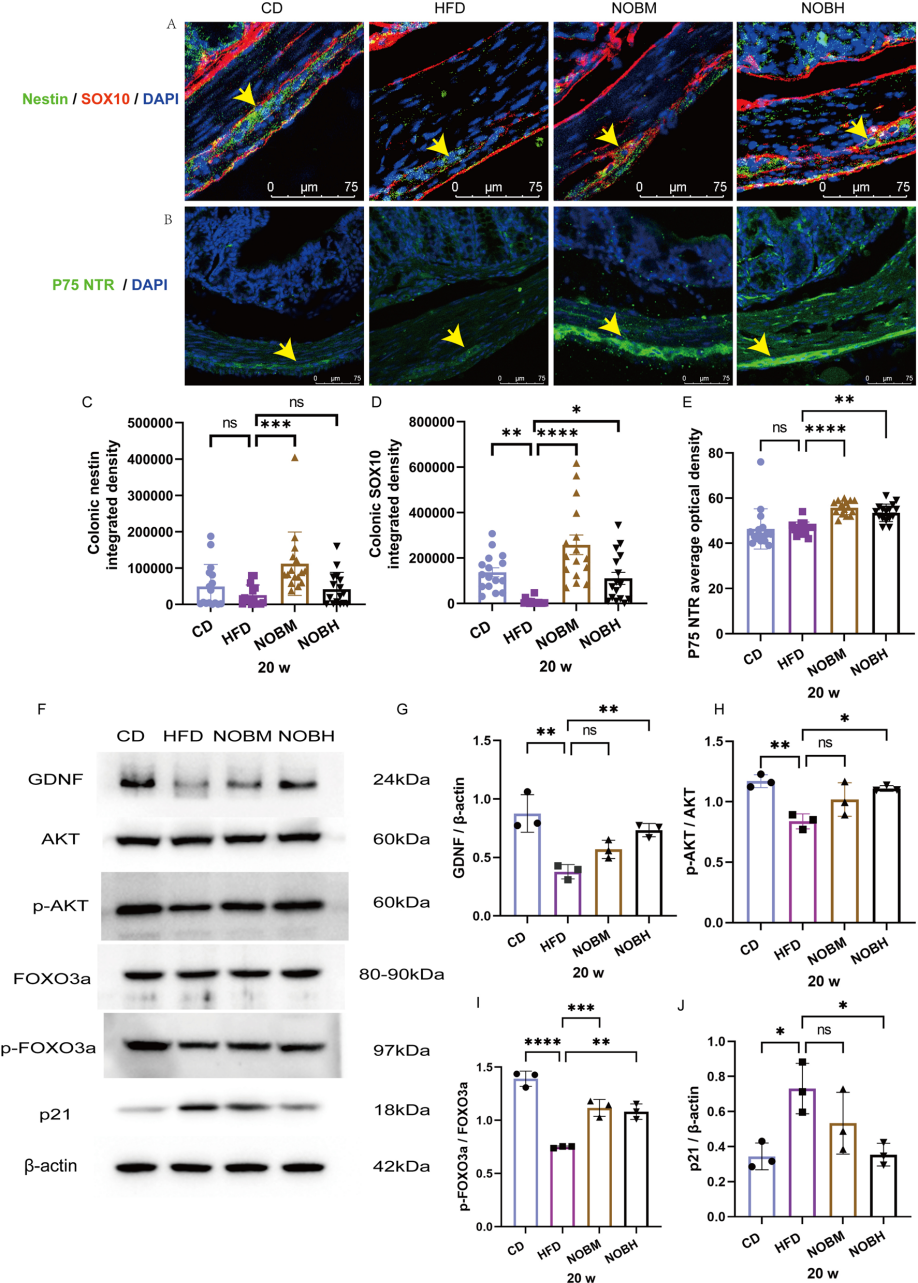
Nobiletin promoted enteric nerve regeneration via the GDNF/AKT/FOXO3a/P21 pathway in HFD-fed mice. **A**,** C** **D** Nestin and SOX10 expression and statistical analyses. **B**, **E** P75NTR expression and statistical analyses. **F** WB quantification of β-actin, GDNF, p-AKT, AKT, p-FOXO3a, FOXO3a and P21 protein expression. **G**–**J** Statistical analyses of GDNF/β-actin, p-AKT/AKT, p-FOXO3a/FOXO3a and P21/β-actin. *P < 0.05, **P < 0.01, ***P < 0.001, ****P < 0.0001, ns > 0.05. **A**–**E** n = 4, each dot in **C**–**E** represents the optical density of the images taken from the upper, lower, left and right portions of the section. **F**–**J**, n = 3. CD: control group; HFD: high-fat diet group; NOBM, 100 mg/kg/d nobiletin; NOBH, 200 mg/kg/d nobiletin. Positive staining is indicated by a yellow arrow

Nestin is a marker of neural progenitor cells. p75 is a neurotrophin receptor (p75NTR). SOX10 is a Schwann cell marker. Nestin, SOX10 and P75NTR are associated with enteric nerve regeneration (Meeker and Williams 2015; Bondurand et al. 2006; Pfeifle et al. 2017). In this study, compared with those in the CD group, nestin and p75NTR expression did not significantly change, which indicates that a HFD has little effect on neural progenitor cells in the colon (Fig. 5A–C and E). However, nobiletin treatment significantly increased nestin expression in the NOBM group compared with that in the HFD group (Fig. 5A, C) (P < 0.001). A HFD induced a significant decrease in SOX10 expression (Fig. 5A, D) (P < 0.01). However, SOX10 expression was significantly greater in the NOBM and NOBH groups than in the HFD group (Fig. 5A, D) (P < 0.0001 and 0.05). Interestingly, SOX10 and Nestin double-positive cells were found in the NOBM and NOBH groups, suggesting that SOX10-positive cells have reserve stem cell potential (Fig. 5A). Compared with that in the HFD group, P75NTR expression was significantly greater in the NOBM and NOBH groups (Fig. 5B, E) (P < 0.0001 and 0.01). To confirm whether nobiletin promoted enteric nerve cell regeneration, EDU was used to assess proliferation in vivo. There were more PGP9.5 and EDU double-positive cells in the NOBM and NOBH groups than in the HFD group at 17 w and 18 w. However, due to the small sample size, no statistical analysis was conducted in this study (Supplementary Fig. 2). In conclusion, nobiletin promoted enteric nerve regeneration.

FOXO3 is associated with the proliferation and differentiation of neural stem cells (Zhang et al. 2020). Next, GDNF/AKT/FOXO3a/P21 pathway-related proteins were detected by WB (Fig. 5F–J). A HFD induced a significant decrease in GDNF expression and the phosphorylation of AKT and FOXO3a and a significant increase in P21 expression. However, compared with the HFD group, nobiletin treatment increased GDNF expression and the phosphorylation of AKT and FOXO3a and decreased P21 expression (Fig. 5F–G). In conclusion, nobiletin-promoted enteric nerve regeneration may be related to the GDNF/AKT/FOXO3a/P21 pathway in obese mice.

### Nobiletin promoted ENSC proliferation and inhibited ENSC apoptosis

**Fig. 6:**
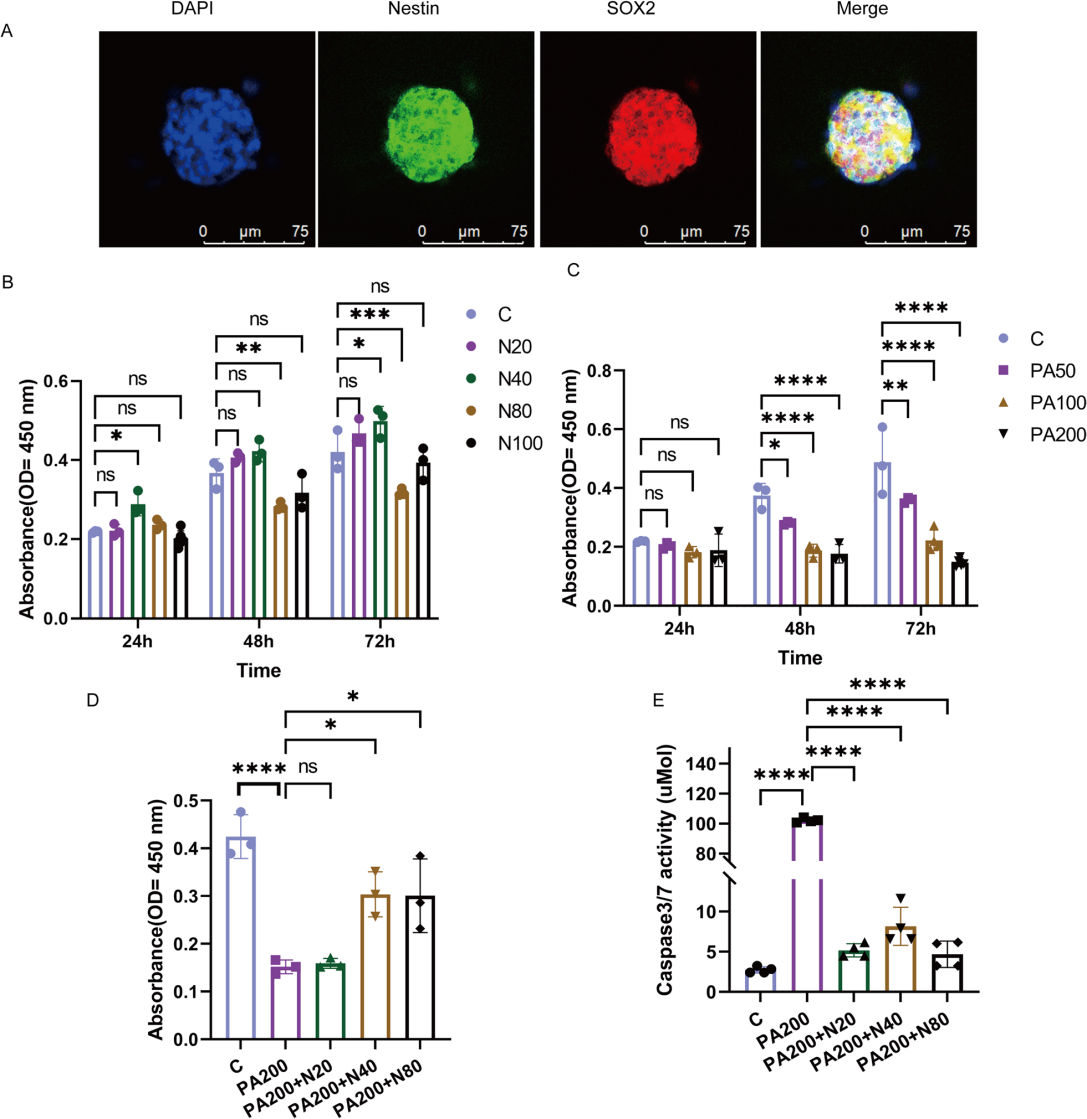
Nobiletin promoted ENSC differentiation and proliferation. **A** The identification of ENSCs. **B**, **C** Cytotoxicity experiment of nobiletin and PA. **D** Nobiletin promoted PA-induced ENSC damage proliferation. **E** Nobiletin decreased caspase3/7 enzymatic activity. *P < 0.05, **P < 0.01, ***P < 0.001, ****P < 0.0001, ns > 0.05. n = 3. C, without PA and nobiletin. N20, 20 μM nobiletin. N40, 40 μM nobiletin. N80, 80 μM nobiletin. N100, 100 μM nobiletin. P200 + N20, PA 200 μM PA + 20 μM nobiletin. PA200 + N40, 200 μM PA + 40 μM nobiletin. PA200 + N80, 200 μM PA + 80 μM nobiletin

To further explore the effects of nobiletin on ENSCs, we cultured primary ENSCs. The expression levels of the neural stem cell markers Nestin and SOX2 are shown in Fig. 6A. Next, cytotoxicity experiments with nobiletin and PA were performed (Fig. 6B and C). Compared with the C group, 20 and 40 μM nobiletin had little or no cytotoxic effect on ENSCs, especially at 40 μM concentration at 72 h (P < 0.05). Compared with those in the C group, 50, 100 or 200 μM PA had cytotoxic effects on ENSCs, especially at a concentration of 200 μM at 72 h (P < 0.0001) (Fig. 6C). Therefore, we selected nobiletin at 20, 40 or 80 μM and PA at 200 μM for the next experiment. Treatment with 40 or 80 μM nobiletin significantly promoted ENSC proliferation in the 200 μM PA group compared with that in the C group (Fig. 6D) (P < 0.05). Compared with the C group, the 200 μM PA group exhibited significantly increased activity of the caspase3/7 enzyme (P < 0.0001). However, compared with those in the PA200 group, nobiletin at 20, 40 or 80 μM significantly decreased the activity of the caspase3/7 enzyme (Fig. 6E) (P < 0.0001). Based on these data, we can infer that nobiletin protected against PA-induced ENSC damage by promoting ENSC proliferation and inhibiting apoptosis.

## Discussion

HFD-induced enteric nerve damage has been reported in previous studies (Bhattarai et al. 2016; Pang et al. 2022). However, there are few effective treatments for HFD-induced enteric nerve damage. Studies have shown that nobiletin can inhibit neuronal apoptosis (Pang et al. 2022; Wang et al. 2019; Zheng et al. 2017) and neuroinflammation (Wang et al. 2019; Zheng et al. 2017) and increase neurotrophic factor expression (Li et al. 2013; Yang et al. 2017). In this study, nobiletin also exerted protective effects on HFD-induced enteric nerve damage.

Nobiletin can improve abnormal glucose tolerance, protect enteric nerves and reduce lipid accumulation. Kim et al. (2021) reported that nobiletin can reduce body weight by increasing brown fat thermogenesis (Kihara-Negishi et al. 2022) and inducing browning of white adipose tissue (Lone et al. 2018). In addition, nobiletin can also reduce weight and promote lipid metabolism by regulating the intestinal flora (Kou et al. 2021). In our previous study (Pang et al. 2022), nobiletin also regulated the expression of leptin and adiponectin to promote lipid metabolism (Pang et al. 2022). A recent study showed that nobiletin can improve HFD-induced impaired glucose tolerance by regulating the biological rhythm of glucagon-like peptide-1 secretion (Martchenko et al. 2022). The lipids that accumulate in intestinal neurons can induce neuroapoptosis through mitochondrial oxidative stress and endoplasmic reticulum stress (Nezami et al. 2014). In this study, more lipid accumulation in the colon of the HFD group was observed. Intestinal lipid accumulation has been reported in a few studies (Ling et al. 2019; Zhao et al. 2021). However, lipid accumulation in the colon has rarely been reported. Intestinal lipid accumulation leads to induced endoplasmic reticulum stress and activated autophagy and apoptosis (Ling et al. 2019). Increased lipid accumulation in the colon may be associated with lipid peroxidation (Pang et al. 2022), inflammation (Pang et al. 2022) and neuroapoptosis (Nezami et al. 2014). In conclusion, nobiletin protected against HFD-induced enteric nerve damage by reducing lipid accumulation and peroxidation.

Nobiletin protected the enteric nerve by regulating Trem2 expression and glial cell activation. Trem2 is an important modulator of the inflammatory response (Wu et al. 2017). Trem2 promotes DAP12 and DAP10 phosphorylation to initiate intracellular signal transduction and activate spleen tyrosine kinase (Syk) and PI3K (Peng et al. 2010). Trem2 also inhibited inflammation through the regulation of TLR expression by DAP12 (Jaitin et al. 2019). Trem2 induces macrophage reprogramming from M1 to M2 to promote phagocytosis and lipid metabolism, promote cell survival, and inhibit inflammation (Deczkowska et al. 2020). Trem2 deficiency can promote the overactivation of microglia and lead to neuroinflammation and neurotoxicity (Kihara-Negishi et al. 2022). However, overexpression of Trem2 in the hippocampus inhibited HFD-induced neuroinflammation and improved cognitive dysfunction (Jaitin et al. 2019). With decreasing Trem2 expression, M1 microglia may increase IL-1β and IL-6 expression and reduce IL-10 expression (Orihuela et al. 2016). Lipopolysaccharide (LPS) activated the TLR4/NF-κB pathway and increased the expression of NOS2, IL-1β and IL-6 by reducing Trem2 expression (Zhang et al. 2019). However, a HFD could increase the LPS concentration (Hersoug et al. 2018), which could explain why Trem2 expression was decreased in the HFD group in this study. Nobiletin increased the expression of Trem2 and decreased the expression of GFAP and NOS2 in this study. Based on the above discussion, nobiletin may inhibit glial cell activation by increasing Trem2 expression. Overactivation of microglia leads to neuroinflammation and neurotoxicity (Kihara-Negishi et al. 2022). However, after treatment with nobiletin, the expression of β-tubulin 3, PGP9.5 and Bcl-2 increased, and that of Bax decreased. In conclusion, nobiletin protected against HFD-induced enteric nerve damage by regulating Trem2 expression and glial cell activation and inhibiting enteric nerve apoptosis.

Nobiletin promotes enteric nerve regeneration. Nobiletin promoted Nestin and P75NTR expression. Nestin is a marker protein of neural stem cells. P75NTR can promote ENSC differentiation and proliferation (Meeker and Williams 2015). P75NTR can bind to GDNF (Barati et al. 2006). SOX10 expression is associated with intestinal colonization of spinal cord-derived neural stem cells (Fujiwara et al. 2022). SOX10 can maintain the proliferation of spinal cord-derived neural stem cells and prevent their premature differentiation (Bondurand et al. 2006). Nobiletin promoted Nestin, P75NTR and SOX10 expression. However, is there true nerve regeneration in the colon? Fortunately, PGP9.5 and EDU double-positive cells were observed in the colon after nobiletin treatment. In summary, the protective effect of nobiletin on HFD-induced enteric nerve damage may be associated with the promotion of enteric nerve regeneration.

Nobiletin may promote GDNF expression by regulating astrocyte polarization. There are two main types of astrocytes: A1 and A2. A1 neurotoxic astrocytes were significantly increased and GDNF was significantly decreased in neurodegenerative diseases such as Parkinson's disease (Garcia-Dominguez et al. 2018). Studies have shown that A2 astrocytes produce GDNF and BDNF (Gurram et al. 2023; Fujita et al. 2018). In this study, GFAP expression was significantly increased and GDNF expression was significantly decreased in HFD-fed mice. Based on the above discussion, we speculated that a HFD induces an increase in the number of neurotoxic A1 astrocytes in the colon. In addition, nobiletin promoted GDNF expression by regulating astrocyte polarization.

Nobiletin promoted colon nerve cell proliferation through the GDNF/AKT/FOXO3a/P21 pathway. GDNF is important for the development and damage repair of the enteric nerve (Heanue and Pachnis 2007). GDNF can activate the AKT pathway (Heanue and Pachnis 2007), and AKT inactivates FOXO3a through phosphorylation of FOXO3a and regulates P21 and P27 expression (Obsilova et al. 2005). In this study, nobiletin promoted GDNF expression and the phosphorylation of AKT and FOXO3a. Phosphorylated FOXO3a (p-FOXO3a) translocated from the nucleus to the cytoplasm, and P21 expression decreased. One study showed that P21 was involved in the proliferation and development of nerve cells (Rajan et al. 2017). When P21 was increased, the proliferation of nerve cells decreased (Rajan et al. 2017). However, after P21 knockout, the number of nerve cells and neural stem cells in the brain significantly increased (Battistini et al. 2023). A proliferation-promoting effect was also observed in ENSCs in this study. These results suggested that nobiletin promotes enteric nerve cell proliferation via the GDNF/AKT/FOXO3a/P21 pathway.

In summary, nobiletin can improve obesity-related indices and abnormal glucose tolerance; reduce lipid accumulation and peroxidation, enteric glial cell activation, nerve injury and apoptosis; and promote enteric nerve regeneration in obese mice.

## Conclusions

In conclusion, nobiletin protected against HFD-induced colonic enteric nerve damage by reducing glial cell activation and enteric nerve apoptosis and promoting enteric nerve regeneration in obese mice. However, key proteins and signalling pathways were not blocked to prove the neuroprotective effect of nobiletin in obese mice, and more in-depth research is needed.

### Supplementary Information

Supplementary Material 1.

## Data Availability

The original contributions presented in the study are included in the article/Supplementary Material, and further inquiries can be directed to the corresponding author/s.

## Abbreviations

GDNF: Glial cell line-derived neurotrophic factor
AKT: Protein kinase B
FOXO3a: Forkhead box O3a
HFD: High fat diet
ENSC: Enteric neural stem cell
PGP9.5: Ubiquitin carboxy-terminal hydrolase L1
EDU: 5-Ethynyl-2′-deoxyuridine
SOX10: SRY-box transcription factor 10
P75NTR: Nerve growth factor receptor
BDNF: Brain-derived neurotrophic factor
ENS: Enteric nervous system
Bcl-2: B-cell lymphoma-2
TNF-α: Tumor necrosis factor 2
IL-1β: Interleukin-1β
NOS2: Nitric oxide synthase 2
LPS: Lipopolysaccharide
TLR4: Toll like receptor 4
NF-κB: Nuclear factor kappa B subunit
Syk: Spleen tyrosine kinase
DAP12: Transmembrane immune signaling adaptor TYROBP
NOBM: Moderate dose of nobiletin
NOBH: High dose of nobiletin
MDA: Malonaldehyde
OGTT: Oral glucose tolerance test
CCK8: Cell counting kit-8
SOD: Superoxide dismutase
GFAP: Glial fibrillary acidic protein
Bax: BCL2 associated X
IL-10: Interleukin 10
IL-6: Interleukin 6
qPCR: Quantitative polymerase chain reaction
