# Supplementary material for: Nobiletin restores HFD-induced enteric nerve injury by regulating enteric glial activation and the GDNF/AKT/FOXO3a/P21 pathway
Source: Mol Med. 2024 Aug 2;30:113. doi: 10.1186/s10020-024-00841-8 (PMC11297793; doi:10.1186/s10020-024-00841-8)
Supplement: Supplementary file 1 — Supplementary Material 1. [file 10020_2024_841_MOESM1_ESM.docx]

**Supplementary Material**

**1.Supplementary Figures**


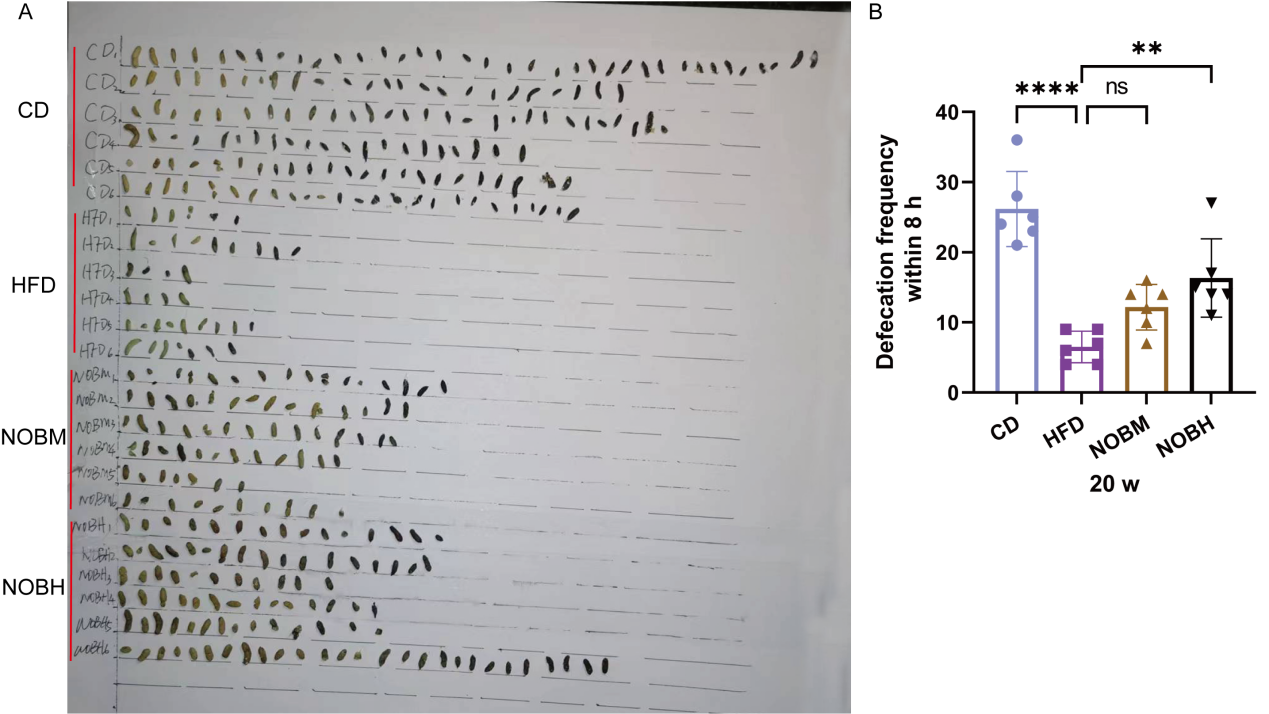


**Supplementary Figure 1. Nobiletin improved the** **defecation frequency and** **colonic pathological score.** **(A)** Defecation frequency of mice in 8 h (n=6). **(B)** Statistical analyses of defecation frequency (n=6). *P<0.05, **P<0.01, ***P<0.001, ****P<0.0001, ns>0.05. CD, control group; HFD, high fat diet; NOBM, nobiletin 100 mg/kg/d; NOBH, nobiletin 200 mg/kg/d.


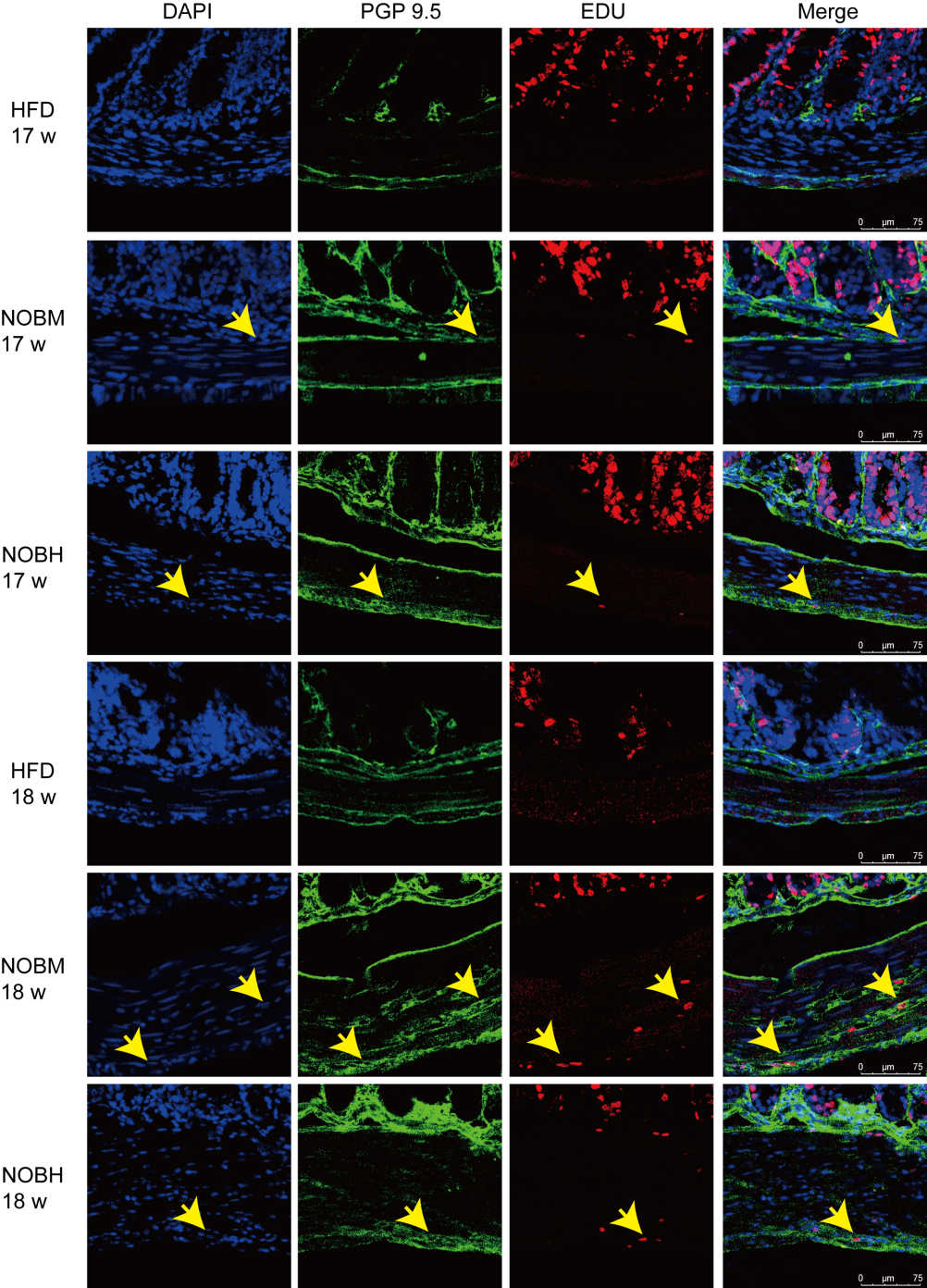


**Supplementary Figure 2. Nobiletin increases EDU and PGP 9.5 expression.** CD, control group; HFD, high fat diet; NOBM, nobiletin 100 mg/kg/d; NOBH, nobiletin 200 mg/kg/d. Positive staining is indicated by yellow arrows. n=1.

**2.Supplementary Tables**

**Supplementary Table 1. Primer sequence**

| Gene | Forward primer | Reverse primer |
| --- | --- | --- |
| β-actin | 5’-ACTGTCGAGTCGCGTCC-3’ | 5’-CTGACCCATTCCCACCATCA-3’ |
| NOS2 | 5’-GGAGATGGTCCGCAAGAGAG-3’ | 5’-GCAAATGTAGAGGTGGCCCT-3’ |
